# Oncology-Inspired Treatment Options for COVID-19

**DOI:** 10.2967/jnumed.120.249748

**Published:** 2020-12

**Authors:** Nagavarakishore Pillarsetty, Lukas M. Carter, Jason S. Lewis, Thomas Reiner

**Affiliations:** 1 Department of Radiology, Memorial Sloan Kettering Cancer Center, New York, New York; 2 Molecular Pharmacology Program, Memorial Sloan Kettering Cancer Center, New York, New York; and; 3 Chemical Biology Program, Memorial Sloan Kettering Cancer Center, New York, New York

**Keywords:** auger, radiotherapy, COVID-19, SARS-CoV-2, CR3022

## Abstract

CR3022 is a human antibody that binds to severe acute respiratory syndrome coronavirus 2 (SARS-CoV-2). Here, we explore the use of CR3022 as a molecularly targeted radiotherapeutic. **Methods:** CR3022 was labeled with ^131^I and purified, yielding ^131^I-CR3022. Using a magnetic bead assay and a recombinant SARS-CoV-2 spike protein fragment, we tested binding of ^131^I-CR3022 in the presence and absence of CR3022. **Results:** We conjugated the antibody CR3022 with a purity of more than 98% and a specific activity of more than 292 MBq/mg. Using a bead-based assay, we confirmed that binding of ^131^I-CR3022 is selective and is significantly reduced in the presence of unlabeled antibody (3.14% ± 0.14% specific uptake and 0.10% ± 0.01% specific uptake, respectively; *P* < 0.0001). **Conclusion:** Our results confirm the potential of CR3022 as a molecularly targeted probe for SARS-CoV-2. A labeled version of CR3022 could potentially be used for Auger radiotherapy or noninvasive imaging.

Radiotherapy, the treatment of disease with ionizing radiation, plays a significant role in the treatment and management of cancer. Most recently, molecularly targeted endoradiotherapeutics have received significant attention (1). These agents consist of a targeted vector (a small molecule, a peptide, or an antibody) and a radioactive payload (an α-emitter, a β-emitter, or an Auger emitter). Some of these treatments have generated impressive responses, such as was seen for ^177^Lu-DOTATATE (Lutathera; Advanced Accelerator Applications), a β-emitter recently approved by the Food and Drug Administration that has a half-life of 6.7 d and extends both progression-free and overall survival in the setting of midgut neuroendocrine tumors (2).

For molecularly targeted endoradiotherapeutics, particular focus has to be placed on the kind of radioactive emitter, as different disintegration pathways produce particle emissions of varying type and profile (3,4). Matching the half-life and decay type to a particular application is therefore imperative and can determine success or failure.

The most recognized radioactive emissions of therapeutic relevance are α- or β-particles, which represent a H24e2+ nucleus and an electron, respectively. α-emitting radioisotopes have particle pathlengths of 50–100 μm and high linear energy transfer (5) rates (80 keV/μm). β-emitting radioisotopes have particle pathlengths of up to several millimeters in soft tissue and significantly lower linear energy transfer rates (∼0.2 keV/μm). With the coronavirus disease 2019 (COVID-19) pandemic in mind, both α- and β-particles are therefore likely suboptimal therapeutics, considering severe acute respiratory syndrome coronavirus 2 (SARS-CoV-2) virions’ diameters (80–120 nM (6)) and the associated potential for detrimental effects on surrounding tissue. However, Auger electron emitters appear to be of particular significance here. Auger electrons combine a relatively high linear energy transfer (4–26 keV/μm) with extremely short nanometer-to-micrometer particle pathlengths, concentrating their cytotoxic potential into minute volumes compared with cellular dimensions. In a rough approximation, Auger electrons with energies of between 0.5 and 10 keV are sufficiently energetic to penetrate deep into the virus, producing direct and indirect radiologic effects (i.e., therapeutic action) when originating at the viral envelope, but are insufficiently energetic to directly damage neighboring cell nuclei.

In oncology, such characteristics led to attempts to incorporate Auger electrons into artificial nucleotides in order to treat cancers by causing complex DNA damage in tumor cells, but this approach has ultimately not been successful because of the difficulty of delivering lethal doses to a large enough fraction of tumor cells within a particular lesion (7–9). Recent preclinical work using different delivery mechanisms suggests a renewed promise and corroborates the considerable toxic effect of Auger emitters on tumor cells (10). In Figure 1, we illustrate the relationship between Auger/conversion electron energy and yield for ^125^I. Examples for other prolific Auger emitters (11) can be found in Figure 2.

**FIGURE 1.**
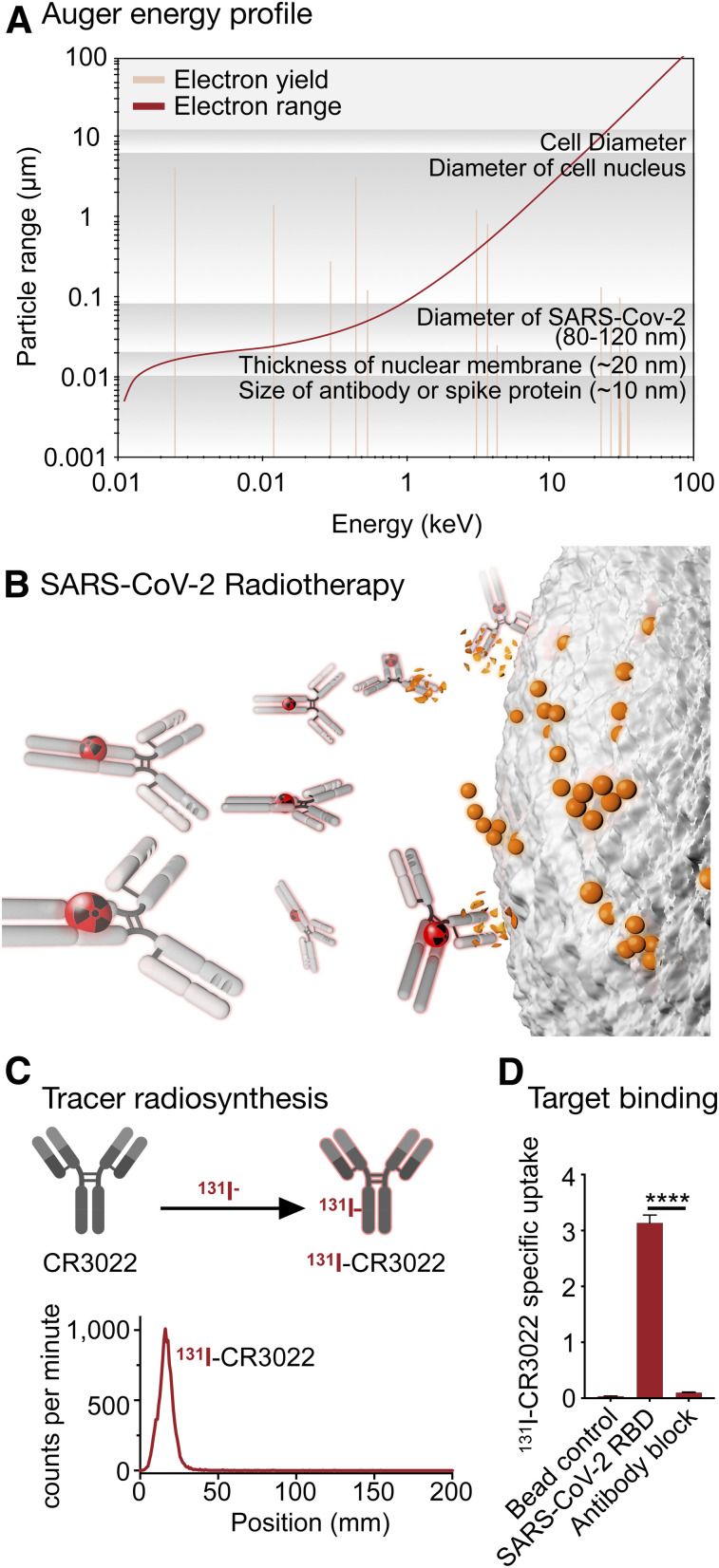
New trick for old dog. (A) Destruction of tumor cells with targeted radioactive isotopes is important in standard-of-care oncology. ^125^I-iodine has energy profile that would allow deposition of energy within radius of SARS-CoV-2 virion. (B) Decay events damage sensitive DNA within tumor cell nucleus, causing catastrophic single- and double-strand breaks. Clinical use of antibody-delivered Auger emitters could open window for targeted destruction of extracellular COVID-19 virions, decreasing viral load during active infection and potentially easing disease burden for patient. (C) Labeling of CR3022 with ^131^I-iodine. (D) Confirmation of specific binding SARS-COV-2 spike protein, subunit S1. ****P < 0.0001, unpaired Student *t* test.

**FIGURE 2.**
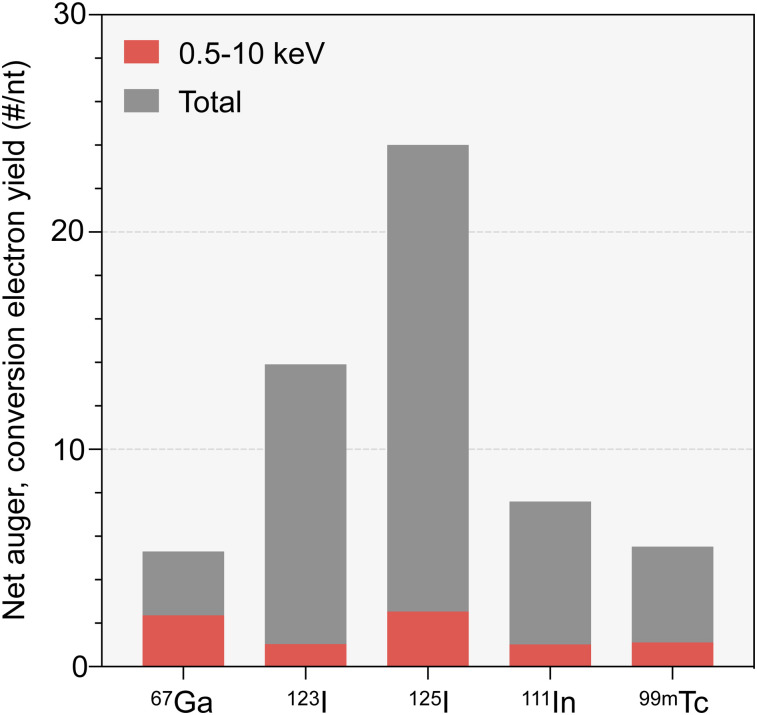
Net yields of monoenergetic electrons (Auger, conversion electrons) per nuclear transformation for ^67^Ga, ^123^I, ^125^I, ^111^In, and ^99m^Tc. Red bars represent contribution, to total yield, of electrons within 0.5- to 10-keV energy range. Yields were obtained from ICRP publication 107 (29).

^125^I is reactor-produced and available in large quantities. At the time of writing, the McMaster nuclear reactor in Hamilton, Ontario, produces the isotope predominantly for brachytherapy, allowing treatment of 70,000 patients annually.

In the past, ^125^I was explored as an Auger-based radiotherapeutic for a genetically engineered measles virus. The virus, which expressed the sodium iodide symporter in infected cells, was sensitive to ^125^I in vitro, where virus replication could be stopped. These results, however, did not translate to an in vivo model, suggesting suboptimal pharmacokinetics of ^125^I-iodide (12). A selective, molecularly targeted vector such as the monoclonal antibody CR3022 could serve as a delivery agent for ^125^I. CR3022 binds to the SARS-CoV-2 receptor binding domain with a dissociation constant of 6.3 nM. The antibody is cross-reactive and conserved across several coronaviruses, making it ideal for targeting not only SARS-CoV-2 but also, potentially, related diseases (13,14).

Another iodine isotope, ^131^I, not only is used as a standard-of-care treatment for certain types of thyroid cancers but also finds widespread use in scintigraphy and whole-body SPECT imaging. Intuitively, a radiolabeled CR3022 could be valuable for imaging, potentially serving as a direct, spatially resolved, contemporaneous, and noninvasive readout of viral load within a patient. From a drug-development perspective, a direct readout of SARS-CoV-2 viral load could represent a quick, upstream indicator of therapy success. This tool could be particularly interesting for clinical research, and similar approaches have been used to accelerate oncologic drug development pipelines in the past (15).

## MATERIALS AND METHODS

### General

All reagents were obtained from commercial vendors and used without further purification. Phosphate-buffered saline (PBS), 0.9%; Iodo-Gen (1,3,4,6-tetrachloro-3α,6α-diphenyl-glycoluril; Pierce Biotechnology, Inc.); and dichloromethane were obtained from Thermo Fisher Scientific. Anti-SARS-CoV-2 antibody CR3022 was purchased from Creative Biolabs. Recombinant SARS-CoV-2 spike protein, subunit S1 (host cell receptor binding domain–receptor binding domain), with an N-terminal histidine tag was purchased from Raybiotech. Magnetic beads 1 μm in diameter and functionalized with HisPur nickel-nitrilotriacetic acid for the bead assay were purchased from Thermo Fisher Scientific. Iodo-Gen–coated glass reaction tubes were prepared by evaporating 50 μL of Iodo-Gen solution (50 μg, 1 mg/mL) in a borosilicate glass test tube (12 × 75 mm). PD MiniTrap G-25 columns (GE Healthcare) were preconditioned with 2 mL of PBS before being used to separate radioiodinated antibody from the free radioiodine.

### Radiosynthesis

A 70-μL volume of PBS was added to an Iodo-Gen (100 μg)–precoated culture tube. To the resulting solution, a 25-μg quantity of CR3022 monoclonal antibody (25 μL, 1.0 mg/mL) was added. A 9.25-MBq (250 μCi) quantity of ^131^I-NaI (in 17 μL of 0.1N NaOH) was added to the solution in the tube, and the mixture was allowed to react for 4 min at room temperature before being loaded onto a PD MiniTrap G-25 column (GE Healthcare) preconditioned with 2 mL of PBS. The radiolabeled antibody was purified using saline as the eluant. Fraction 3 was used for the binding studies. The purity of the compound was measured using silica gel instant thin-layer chromatography paper, with 10% trifuloroacetic acid in water as the eluent. The specific activity of the final product was 292 MBq/mg (7.9 mCi/mg; 177.5 μCi/22.5 μg).

### Magnetic Bead Assay

We have previously described the details of the bead-based assay (16). The assay comprises 3 separate arms—a control with no antigen, a positive control, and a blocking control—and each arm analyzes samples in triplicate. The first arm serves as a control to measure nonspecific binding of the radioligand to the beads without any target antigen (SARS-CoV-2 spike protein, subunit S1), the second arm assesses radioligand binding to beads coated with this antigen, and the third arm validates the specificity of radioligand binding to the cognate antigen in the presence of an excess of unlabeled ligand.

Briefly, samples were prepared by aliquoting 20 μL of the magnetic bead slurry into a 1.5-mL LoBind microcentrifuge tube (Fisher Scientific). The beads were washed by adding 380 μL of PBS containing 1% bovine serum albumin. The tubes were placed in a vortex mixer for 5 s, followed by a brief spin in a mini-centrifuge before being placed on a magnetic rack (DynaMag-2; ThermoFisher Scientific) for 30–45 s to isolate the magnetic beads. The SARS-CoV-2, subunit S1, antigen was resuspended to achieve a concentration of a 0.1 mg/mL. The washed beads were resuspended in 390 μL of PBS–bovine serum albumin, and the beads in all tubes except the control arm were incubated with 1 μg (10 μL) of His-tagged or biotinylated antigen for 15 min on an Eppendorf Thermomixer at 300 rpm at room temperature. Subsequently, the beads were washed once with 400 μL of PBS-T before the addition of 1 ng of the radiolabeled antibody (^131^I-CR3022) and resuspension in 1% bovine serum albumin–PBS. ^131^I-CR3022 was incubated with antigen-coated beads for 30 min on a rotating mixer at room temperature. A large excess (5 μg) of the unlabeled cold antibody CR3022 was added a few seconds before 1 ng of the radioligand was added to antigen-coated beads in the blocking arm. Thereafter, the beads were isolated using a magnet, and the supernatant containing unbound radioligand was aspirated with a pipette and collected in separate tubes. To remove nonspecifically bound radioligand, the beads were washed twice with 400 μL of PBS-bovine serum albumin. Finally, the beads, supernatant, and washes were measured for radioactivity on a γ-counter. The relative binding fractions were determined by dividing the percentage of total activity bound to magnetic beads to the total activity (beads + supernatants + wash).

## RESULTS AND DISCUSSION

SARS-CoV-2 is a coronavirus that emerged in late 2019 and has resulted in an ongoing pandemic, causing cases of COVID-19 across the globe. Common symptoms include fever, cough, shortness of breath, and muscle aches (17). SARS-CoV-2 enters host cells via the angiotensin-converting enzyme 2 receptor, which is expressed in type II alveolar cells of the lungs, and can severely affect lung function (18,19).

Intuitively, many lessons learned from attempts to treat tumors with Auger emitters could be adapted for radiotherapeutically inactivating extracellularly circulating SARS-CoV-2 in patients. After all, tumor cells and SARS-CoV-2 share an important hallmark in their ability to evade patients’ immune systems (20,21). However, whereas tumor cells may permanently escape the immune system (or only become tolerant over the course of the disease), pathogens such as SARS-CoV-2 can be efficiently eliminated once adaptive immunity has been acquired (22). Although it is unlikely that treatment of SARS-CoV-2, mediated by a radiotherapeutic Auger emitter, can eliminate all virions, radiotherapy could be used in combination with other treatments and consequently improve outcomes (Fig. 1). Such treatment combinations could include currently tested treatments such as anti-IL-6 antibodies or remdesivir. However, radiation therapy was reported to initiate and influence the inflammatory and immune system (23), and care has to be taken that there is no subsequent increase in the likelihood of cytokine storms (24).

As a proof of the concept that molecular targeting of SARS-CoV-2 is possible, we turned to CR3022, a human IgG1 antibody constructed from RNA, which was isolated from the lymphocytes of a convalescent SARS-CoV patient from Singapore (25). Although CR3022 is therefore a potent binder of the SARS-CoV-2 receptor binding domain, the recognized epitope does not overlap the angiotensin-converting enzyme 2 binding site (the receptor binding motif), and CR3022 consequently does not compete with angiotensin-converting enzyme 2 for binding to SARS-CoV-2. This characteristic is notably not a drawback for Auger radiotherapy of SARS-COV-2. Using Iodo-Gen for iodination, a method established both in preclinical and clinical settings (26,27), we covalently conjugated ^131^I to commercially available CR3022 with a purity of more than 98% and a specific activity of 292 MBq/mg (Fig. 1C). We confirmed that the modified ^131^I-CR3022 retained its potent binding to SARS-CoV-2 using a magnetic bead assay, testing its binding to a recombinant His-tagged SARS-CoV-2 receptor binding domain.

The specific binding of ^131^I-CR3022 alone was significantly higher without preincubation of unlabeled CR3022, confirming both that CR3022 binds to SARS-CoV-2 and that binding is not perturbed after covalent modification with ^131^I (3.14% ± 0.14% and 0.10% ± 0.01% specific uptake for ^131^I-CR3022 and CR3022, respectively; *P* < 0.0001; Fig. 1D). We consider this experiment a potent first step toward translating an orthogonal therapeutic approach for SARS-CoV-2, which could potentially be used as a combination therapy or monotherapy for patients with active infection. The translational hurdles for such a drug could be lower than with traditional therapeutics or vaccines because the pharmacokinetics (which are dictated by the antibody) are decoupled from the pharmacodynamics (dictated by the radioisotope). Although both work synergistically, they can be optimized separately, similar to what has been done for ^177^Lu-PSMA and ^225^Ac-PSMA, two anticancer radiotherapeutics (28). Substitution of the isotope preserved the pharmacokinetic profile while simultaneously showing therapeutic efficacy in patients with acquired resistance to ^177^Lu-PSMA.

Lastly, the integration of other, previously oncologically deployed, strategies could lead to the rapid rollout of SARS-CoV-2 therapeutics as well, including the conjugation of drug conjugates for treating affected cells, or known antigens for efficiently decloaking SARS-CoV-2 from the immune system.

## CONCLUSION

Our preliminary data, in combination with the available literature, suggest further development of a radiotherapeutic CR3022, which would be merging different pharmacologic approaches.

## DISCLOSURE

This work was supported by National Institutes of Health grants R01 CA204441, R35 CA232130, and P30 CA008748. No other potential conflict of interest relevant to this article was reported.

**KEY POINTS**

**QUESTION:** Can the human monoclonal antibody CR3022 be used as a specific targeted vector for shuttling activity to SARS-CoV-2 virions?
**PERTINENT FINDINGS:** Labeling of CR3022 is possible, and binding affinity of the antibody for the SARS-CoV-2 receptor binding domain is retained.
**IMPLICATIONS FOR PATIENT CARE:** CR3022, modified with a radiolabel, could be used for direct imaging of SARS-CoV-2 but also potentially as an Auger radiotherapeutic in patients with active infection.
